# The Importance of BHB Testing on the Post-Mortem Diagnosis of Ketoacidosis

**DOI:** 10.3390/biom12010009

**Published:** 2021-12-21

**Authors:** Stina Ahlström, Johan Ahlner, Anna K. Jönsson, Henrik Green

**Affiliations:** 1 Department of Forensic Medicine, National Board of Forensic Medicine, 751 40 Uppsala, Sweden; 2 Division of Clinical Chemistry and Pharmacology, Department of Biomedical and Clinical Sciences, Faculty of Medicine and Health Sciences, Linköping University, 582 25 Linkoping, Sweden; johan.ahlner@liu.se (J.A.); anna.jonsson@rmv.se (A.K.J.); henrik.green@liu.se (H.G.); 3 Department of Forensic Genetics and Forensic Toxicology, National Board of Forensic Medicine, 587 58 Linkoping, Sweden

**Keywords:** beta-hydroxybutyrate, ketoacidosis, alcoholism, diabetes, post-mortem, hypothermia, acidosis, autopsy, forensic

## Abstract

Although beta-hydroxybutyrate (BHB) analysis has proved its importance in forensic pathology, its effects on cause-of-death diagnostics are unaddressed. Therefore, this study aims at evaluating the effects of BHB analysis on the number of deaths by DKA (diabetes ketoacidosis), AKA (alcoholic ketoacidosis), HHS (hyperosmolar hyperglycaemic state), hypothermia, diabetes, alcoholism, and acidosis NOS (not otherwise specified). All 2900 deaths from 2013 through 2019 in which BHB was analysed at the National Board of Forensic Medicine, and 1069 DKA, AKA, HHS, hypothermia, diabetes, alcoholism, and acidosis cases without BHB analysis were included. The prevalence of BHB-positive cases for each cause of death, and trends and proportions of different BHB concentrations, were investigated. The number of BHB analyses/year increased from 13 to 1417. AKA increased from three to 66 and acidosis from one to 20. The deaths from alcoholism, DKA, and hypothermia remained stable. It is unclear why death from alcoholism remained stable while AKA increased. The increase in unspecific acidosis deaths raises the question why a more specific diagnosis had not been used. In conclusion, BHB analysis is instrumental in detecting AKA and acidosis. The scientific basis for the diagnosis of DKA and hypothermia improved, but the number of cases did not change.

## 1. Introduction

### 1.1. Background

Beta-hydroxybutyrate (BHB) is a ketone body which is used as an indicator for a metabolic disturbance, ketosis, which develops due to an insufficient intake of carbohydrates. At low concentrations, ketone bodies such as BHB, are well tolerated, but high concentrations lead to ketoacidosis, which may lead to death [1].

Metabolic disorders are frequent among the non-traumatic causes of death encountered in forensic medical practice [2,3]. Analysis of BHB enables the diagnosis of alcoholic ketoacidosis (AKA) post mortem [4,5]. It has also proved important in the diagnosis of diabetes and related acute complications [6] and in the diagnosis of hypothermia [7].

The focus of this previous forensic pathological research has been on the usefulness of BHB analysis for determining the cause of death in various conditions or for diagnosing ketoacidosis [4,8,9,10,11,12,13,14,15]. What previous studies have not addressed is the overall effect the introduction of BHB analysis has had on the cause-of-death diagnostic. How has the number of ketoacidotic diagnoses and related conditions changed in the post-mortem context after BHB analysis was introduced? When a method that enables a new or refined type of diagnosis is set up, this ought to have a diminishing effect on some other cause of death. It should also allow for more specified diagnoses to be used.

### 1.2. Aim

The aim of this study was to compare variations in the number of deaths from diabetes ketoacidosis (DKA), hyperosmolar hyperglycaemic syndrome (HHS), alcoholic ketoacidosis (AKA), hypothermia, unspecified diabetes, chronic alcoholism, and unspecified acidosis (acidosis NOS) in the Swedish forensic autopsy material during the years 2013 through 2019. We also wanted to study any changes in the proportion of positive, ketoacidotic, and negative results as well as determine the most frequent non-acidotic causes of death among the ketoacidotic results.

### 1.3. AKA and Chronic Alcoholism

Before BHB analysis was introduced, AKA was a virtually unknown diagnosis in autopsy practice [4,5]. Therefore, our assumption was that the number of AKAs would increase during the period studied. Furthermore, it was expected that the number of deaths by unspecified chronic alcoholism would decrease since death by chronic alcoholism before the introduction of BHB analysis would have included cases of undiagnosed AKA.

### 1.4. DKA, HHS and Unspecified Diabetes

Analysis of BHB has improved the possibilities of diagnosing DKA and of differentiating between DKA and hyperosmolar hyperglycaemic state (HHS). Previously, DKA could only be diagnosed based on an augmented glucose concentration in vitreous humour, possibly in combination with other suggestive findings, such as an elevated acetone concentration [6]. The diagnosis of hyperglycaemia required vitreous humour to be available. As glucose is unstable post mortem, there is always a risk of falsely negative glucose analyses. Acetone may also be elevated due to reasons other than DKA [6]. Thus, without BHB analysis, it is not possible to reliably determine whether an elevated glucose concentration is due to DKA or HHS. Moreover, the possibility of falsely negative vitreous glucose analyses, or lack of vitreous humour altogether, increased the risk of missing hyperglycaemia. In Swedish forensic autopsy practice, unspecified diabetes is usually classified as ‘diabetes with coma’, i.e., DKA. Thus, even though BHB is important in the diagnosis of DKA, we assumed that the number of deaths from DKA in the Swedish material would remain fairly stable or only slightly increase, even after the introduction of BHB analysis. We assumed that the number of HHS cases would increase since prior to the introduction of BHB analysis, an elevated vitreous glucose concentration would have been considered a sign of DKA. Similarly to chronic alcoholism, the assumption was that the number of deaths from unspecified diabetes would decrease, as BHB analysis provided a new opportunity to diagnose DKA.

### 1.5. Hypothermia

BHB has also been of importance in the investigation of hypothermia. There are some classical morphological signs of hypothermia, such as frost erythema and Wischnewski’s spots, but these are unspecific. An elevated blood BHB concentration is now a widely accepted marker for hypothermia, although a normal concentration of BHB does not exclude hypothermia [7]. Thus, as the diagnosis of hypothermia can be made without BHB analysis, our assumption was that the number of deaths from hypothermia would remain fairly stable or slightly increase. However, the diagnosis of hypothermia would be better validated with ancillary BHB analysis.

### 1.6. Acidosis NOS

Ketoacidosis may arise in a number of other conditions besides chronic alcoholism, diabetes and hypothermia. These include starvation, hypoxia, and multiple organ failure [12,16,17]. Before the introduction of BHB analysis, there may have been sporadic cases in which the cause of death was marked as acidosis. In these cases, the diagnosis would have been based on an elevated acetone concentration. We assumed that with BHB analysis, more of these cases would be detected and the number of acidosis NOS diagnoses would thus increase.

## 2. Materials and Methods

All cases analysed for BHB within the National Board of Forensic Medicine during the years 2013 through 2019 were included in this study. Preferably, femoral blood is used for BHB analysis, but if this is unavailable, another type of blood, such as heart blood, is analysed. BHB is considered stable in blood by several authors [4,9,14,15]. However, the post-mortem interval might affect the measurement, and pericardial fluid has been suggested as an alternative to blood for analysis of BHB [18,19]. The metabolome has also in general been suggested to be affected by the post-mortem interval [20]. The analysis method used has previously been described in Ahlström et al. [21].

The BHB cases were selected by retrieving the reported BHB results from the database of the National Board of Forensic Medicine. In addition, the cases in which the cause of death was either diabetes, chronic alcoholism, hypothermia, or acidosis NOS, but in which BHB was not analysed, were included in the study. These were retrieved from the database of the National Board of Forensic Medicine from the same time period by searching the death certificates for the terms ‘diabetes’, ‘alcohol’, ‘HHS’, ‘hypothermia’, and ‘acidosis’, and including those in which any of these was marked as underlying cause of death. Since all cases from the relevant time period were retrieved, there was no standardisation for post-mortem interval, as the time of death was not known in most cases, which was a weakness of the study.

All these cases were grouped according to cause of death as either DKA, AKA, HHS, hypothermia, acidosis NOS, diabetes NOS, or chronic alcoholism. The grouping was made according to the cause of death that the forensic pathologist had marked on the death certificate in each individual case, based on his/her assessment of the autopsy findings, background information, and results from ancillary investigations. The cases in which BHB was analysed but the cause of death was something else than any of the before-mentioned diagnoses were grouped as ‘others’. As chronic alcoholism were considered cases in which the cause of death was marked either as unspecified chronic alcoholism, or a as chronic disease defined as specifically alcohol related, such as alcoholic liver disease or alcoholic cardiomyopathy, without a specified terminal cause of death. As diabetes NOS were considered cases in which the diabetes was not defined as diabetic ketoacidosis or diabetic coma or HHS. As acidosis NOS cases were considered cases in which the underlying reason for the acidosis was either not defined by the forensic pathologist or its cause was considered by the forensic pathologist to be something else than diabetes, alcohol or hypothermia. All cases included in the study were then separated according to year of analysis and status as BHB analysed or not analysed.

The proportion of cases analysed for BHB that were considered to have an acidotic cause of death (AKA, DKA, hypothermia, or acidosis NOS) were noted separately for each year of the study period. The proportion of BHB concentrations categorised as normal (BHB < 50 μg/g) slightly elevated (BHB 50-249), and pathologically elevated (BHB ≥ 250 μg/g) were noted for each year.

The cases with a BHB concentration ≥ 250 μg/g were further studied. The proportion of cases with a BHB concentration ≥ 250 μg/g and with an acidotic cause of death was noted for each year. The cases with a BHB concentration ≥ 250 μg/g that did not have an acidotic cause of death were grouped separately according to cause of death. The ten most frequent causes of death among these were noted.

## 3. Results

During the years 2013 through 2019, in total 2900 BHB analyses were conducted. During the same time, there were 1069 cases in which the cause of death was marked by the forensic pathologist as DKA, AKA, HHS, hypothermia, acidosis NOS, diabetes NOS, or chronic alcoholism, but in which BHB was not analysed.

The results of the groupings of the cases according to cause-of-death diagnosis are presented in Table 1. The cases without BHB analysis are presented in Table 2 and the combination of these groups in Table 3.

**Figure 1:**
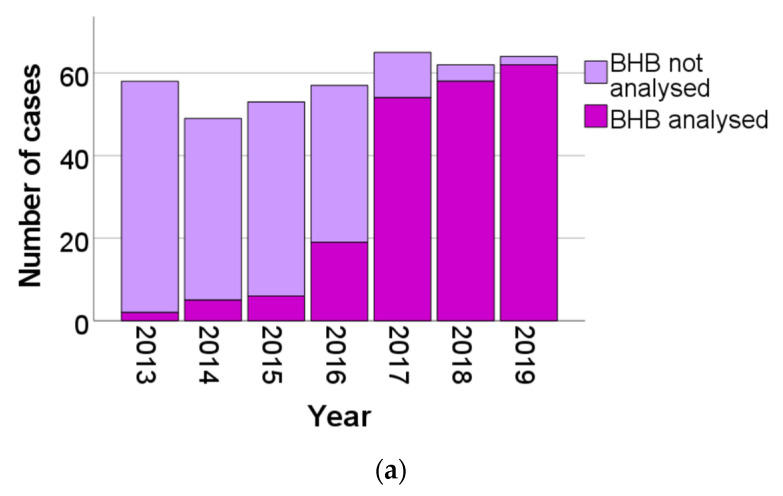

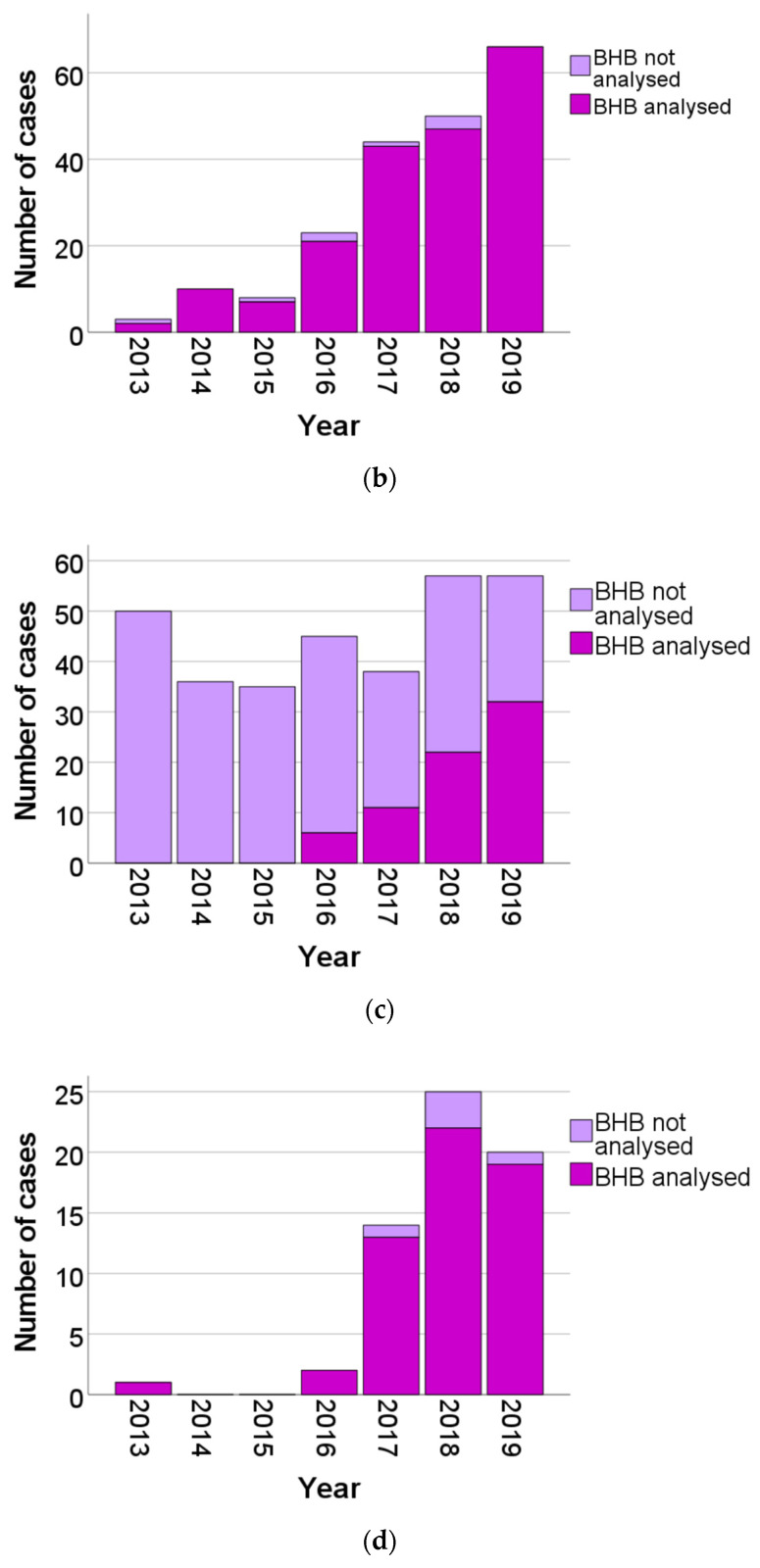
(**a**) Number of DKA (diabetes ketoacidosis) cases, separated as BHB analysed or not analysed. (**b**) Number of AKA (alcoholic ketoacidosis) cases, separated as BHB analysed or not analysed. (**c**) Number of hypothermia cases, separated as BHB analysed or not analysed. (**d**) Number of cases of acidosis NOS (not otherwise specified) per year, separated as BHB analysed or not analysed.

The total number of cases and the proportion of cases per year analysed for BHB and with the cause of death marked as either DKA, AKA, hypothermia, or acidosis NOS are presented in Figure 1. The total number of DKA cases and hypothermia cases per year were fairly stable over the years, although the proportion of cases analysed for BHB increased. Especially, the proportion of analysed DKA cases increased. In 2013, only two cases of DKA were analysed for BHB whereas 56 were not, i.e., only 3.4% of the total number of DKA cases were analysed. By 2019, the proportions were inverse: 62 cases of DKA were analysed for BHB and only two cases were not, i.e., only 3.1% of the total number of DKAs was not analysed. The proportion of hypothermia cases that was analysed for BHB increased as well, but not to the same extent. In 2013, there were in total 50 cases of death by hypothermia, none of which was analysed for BHB. In 2019, there was a total of 57 cases of death by hypothermia, of which 32 cases, i.e., 56%, were analysed for BHB. The AKA cases increased from two analysed cases, and one not analysed case in 2013 to 66 cases in 2019, all of which were analysed for BHB. The number of deaths by acidosis NOS increased from only one case in 2013 to 19 in 2019. These acidosis NOS cases increased especially in 2017 and 2018, and in 2019, there was even a small decrease compared to the previous year.

**Figure 2:**
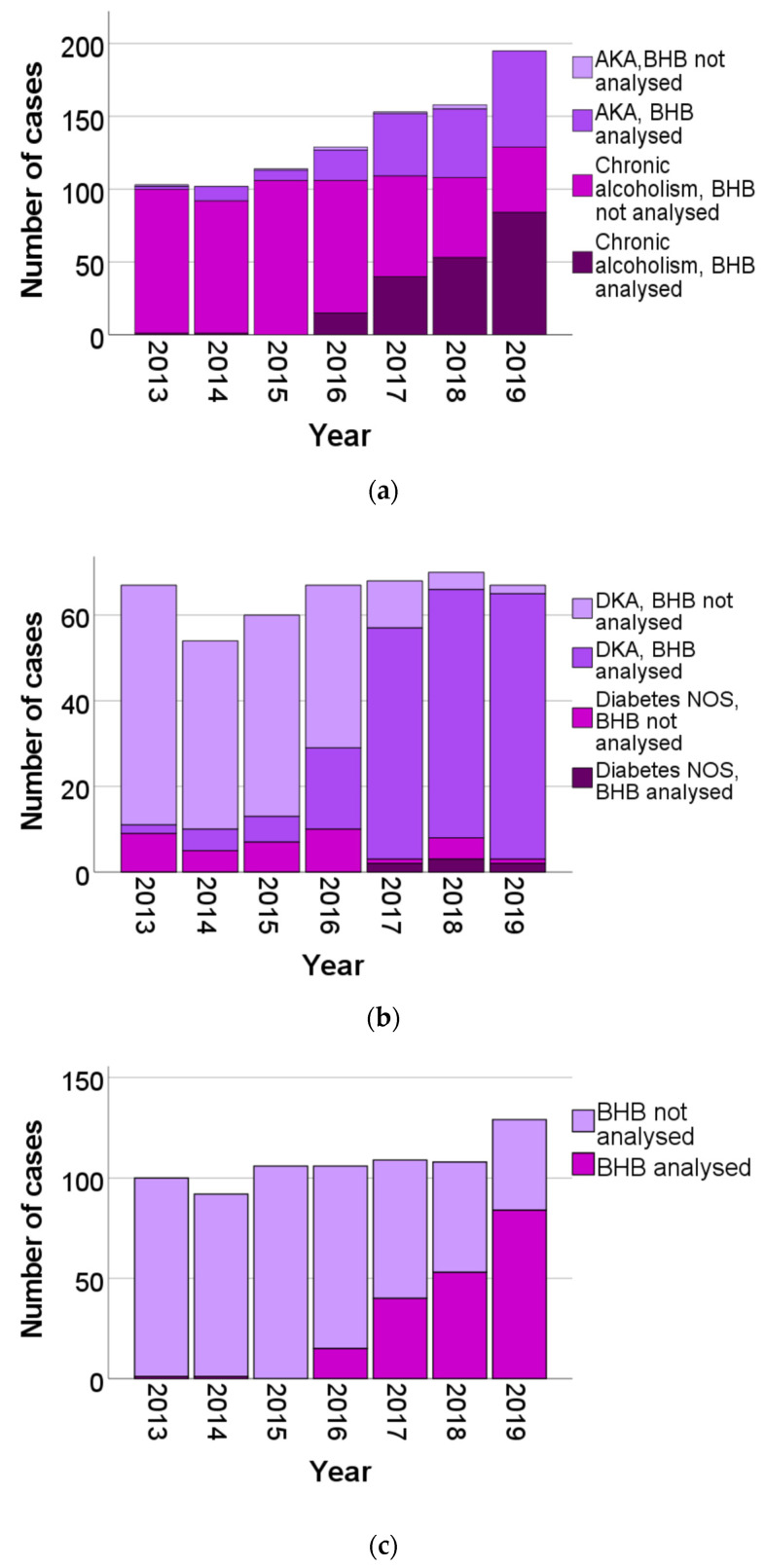

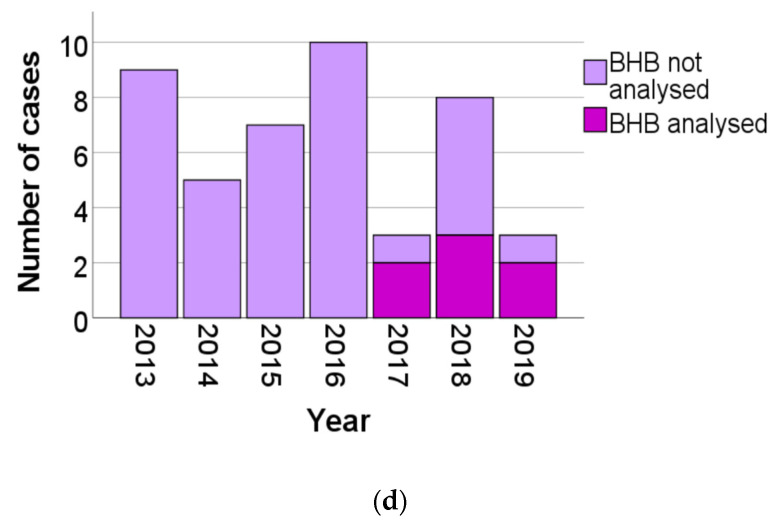
(**a**) Number of AKA (alocoholic ketoacidosis) and chronic alcoholism cases, separated as BHB analysed or not analysed. (**b**) Number of DKA (diabetes ketoacidosis) and diabetes NOS (not otherwise specified) cases, separated as BHB analysed or not analysed. (**c**) Number of chronic alcoholism cases, separated as BHB analysed or not analysed. (**d**) Number of diabetes NOS (not otherwise specified) cases, separated as BHB analysed or not analysed.

Figure 2 presents the combined number of AKA and chronic alcoholism cases, and DKA and diabetes NOS cases per year as well as the proportion of cases analysed for BHB. Figure 2a,c show that, even though AKA increased from three cases in 2013 to 66 cases in 2019, the number of chronic alcoholism cases remained about the same each year or even increased somewhat during the study period. Thus, in spite of the increase in AKA, no decline in chronic alcoholism cases was seen. Regarding diabetes, the number of diabetes NOS cases remained sparse during the whole study period. Hence, the influence of BHB analysis on diabetes NOS could not be determined.

**Figure 3:**
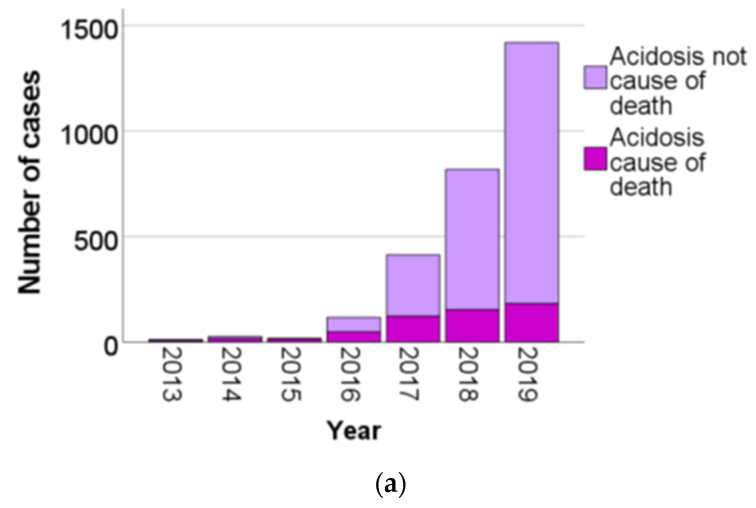

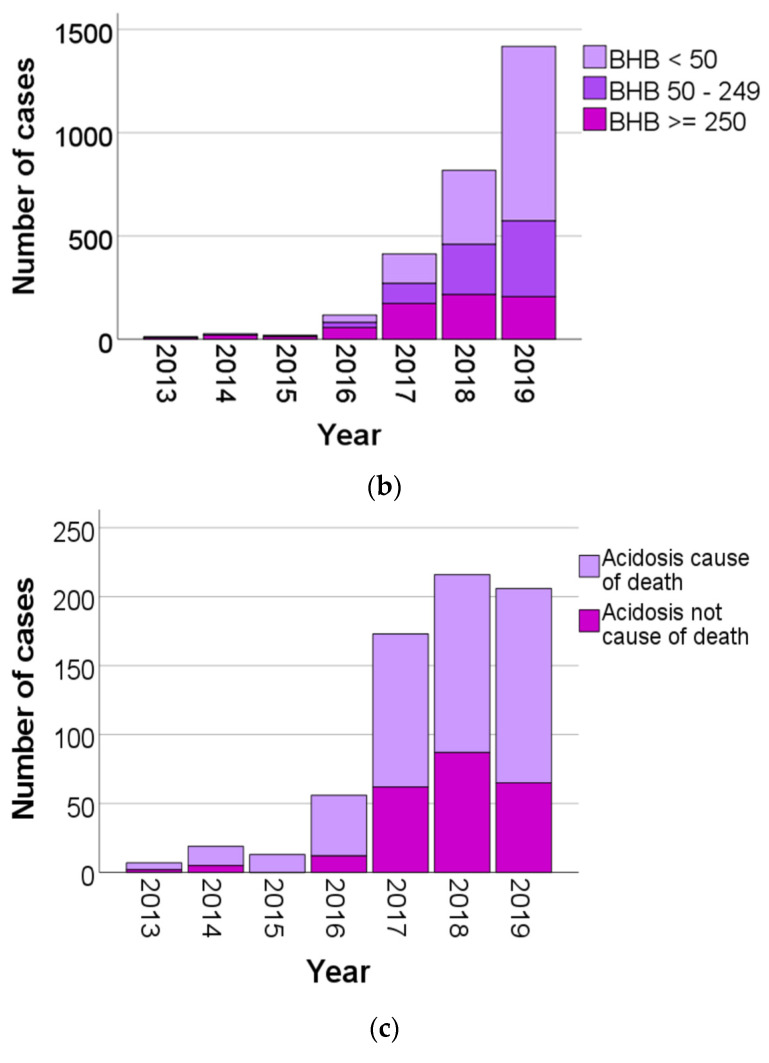
(**a**) Number of BHB analyses per year, separated as acidosis cause or not cause of death. (**b**) Number of BHB analyses per year, separated according to BHB-result category. (**c**) Number of cases with BHB ≥ 250 μg/g per year, separated as acidosis cause of death or not cause of death.

Figure 3 presents the number of BHB analyses for each study year. The proportion of cases with an acidotic cause of death and the proportion with a non-acidotic cause of death are shown in Figure 3a. Figure 3b presents the proportion of various BHB result categories for each year. Figure 3c also presents the proportion of cases with an acidotic cause of death and non-acidotic cause of death in the cases with a BHB concentration ≥ 250 μg/g.

**Figure 4:**
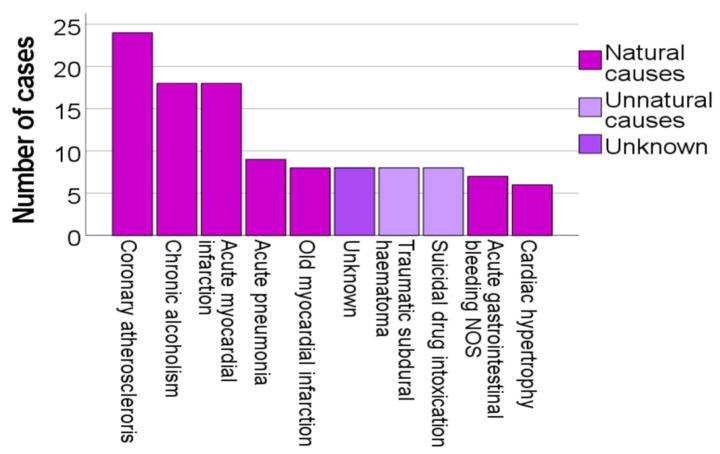
Ten most frequent non-acidotic causes of death when BHB ≥ 250 μg/g.

The ten most frequent non-acidotic causes of death among the cases with a BHB concentration ≥ 250 μg/g are presented in Figure 4 and further separated according to natural, unnatural and unknown causes. Four of these diagnoses were heart diseases (coronary atherosclerosis, acute and old myocardial infarction, and cardiac hypertrophy). Chronic alcoholism was the second most frequent diagnosis. Two diagnoses were unnatural (traumatic subdural haematoma and suicidal drug intoxication), and seven diagnoses were natural. Unknown causes were the sixth most frequent diagnosis. Four diagnoses were of a chronic nature (coronary atherosclerosis, chronic alcoholism, old myocardial infarction, and cardiac hypertrophy).

## 4. Discussion

The results show a large increase in the number of BHB analyses during the study period from 13 in 2013 to 1417 in 2019. This increase in analyses is reflected in all ketoacidotic diagnoses in that an increasing proportion of these diagnoses has been analysed for BHB. AKA and acidosis NOS as causes of death increased dramatically during these years. The number of deaths from chronic alcoholism, DKA, and hypothermia remained stable during the period, but the proportion of BHB positive cases increased.

As mentioned earlier, it was only with the introduction of BHB analysis that the diagnosis of AKA became routine in forensic pathology. This is reflected in our results, too. During the years 2013 to 2015, when only between 13 and 28 analyses in total were conducted per year, the number of AKAs was also very low, between three and ten cases each year. When the number of analyses started increasing from 2016 onwards, so did the number of AKAs. In 2016, 24 cases of AKA were diagnosed, in 2017 there were 44, and in 2018 54. In 2019, there were 66 cases of AKA, which constitutes a 22-fold increase from 2013.

The frequency of AKA, of course, would depend not only on the availability of BHB analysis, but on the frequency of alcoholism in the autopsy population as well. Pounder et al. estimated that AKA was present in about 10% of the chronic alcoholics who were subject to a medico-legal autopsy [22]. In a study by Keltanen et al., 54% of the alcohol abusers in their study had an elevated (>1 mM) total ketone body concentration. These were all cases in which the cause of death was some form of chronic alcoholism, i.e., the number did not include alcoholics with a non-alcoholic cause of death [23]. In comparison, in our material, there were 66 cases of AKA and 84 cases of chronic alcoholism in 2019 among the cases analysed for BHB. This means that in our study, in 2019, 44% (66/(66 + 84)) of cases of direct alcoholic deaths (excluding alcohol poisonings) that were analysed for BHB were cases of lethal AKA. Similarly to the study by Keltanen et al., our figures only include cases of death from chronic alcoholism whereas alcoholics who were subject to a forensic autopsy but had an unrelated cause of death were not included. In 2019, there were also 45 cases of death from chronic alcoholism that were not analysed for BHB. Had these cases been analysed for BHB, the figure of 44% might have been higher.

In Swedish forensic pathology practice, ‘chronic alcoholism’ is often marked as cause of death in cases in which a person has suffered from long-time alcohol abuse and has related organ changes, such as fatty liver or liver cirrhosis, but no specific terminal cause of death can be found. Our assumption was that the number of deaths by such undefined chronic alcoholism would decrease with the increased use of BHB analysis. Instead, the number of deaths from undefined chronic alcoholism remained more or less stable, around 100 cases per year, even though a larger proportion of these was analysed for BHB each year. During the same time, the number of AKA cases increased year by year. Thus, the AKA cases added to the alcoholic deaths in such a manner that total the number of deaths related to chronic alcoholism in the Swedish forensic autopsy material almost doubled from 2013 to 2019.

The reasons behind the stability of the number of deaths from unspecified chronic alcoholism are not entirely clear. The number of forensic autopsies during this time has remained fairly constant (around 5500 autopsies per year) [24]. Thus, it does not seem that the autopsy rate among chronic alcoholics would have changed. However, the expected decrease in death from unspecified chronic alcoholism was not seen. A possible explanation might be that with the arrival of BHB analysis, forensic pathologists are more prone to consider deaths among chronic alcoholics to have been caused by unspecified chronic alcoholism, after they have ruled out AKA. Previously, when AKA was not diagnosable or recognised in the same manner, they may have been more inclined, when possible, to choose a competing cause of death to chronic alcoholism, for example a heart disease.

In contrast to alcohol-related deaths, the number of diabetic deaths (DKA, HHS, and diabetes NOS) during this time remained stable, around 70 cases per year. Indeed, what did change was the proportion of diabetic deaths that was analysed for BHB. In 2013, only a handful of DKAs were analysed for BHB. In 2019, 96% of the diabetic cases (DKA, HHS, and diabetes NOS) were analysed for BHB. The importance of BHB testing in diabetic cases was thus primarily quality-related: the diagnosis of death by DKA became more reliable compared to previously when the diagnosis was made based only on vitreous glucose values, acetone concentration, and health records. A high quality in the establishment of a natural cause of death is important in forensic pathology, as it aids in excluding unnatural causes.

The HHS cases were sparse during the entire study period with the number of cases varying between zero and four each year. As the cases were so few, no observations or conclusions can be drawn. However, the observation that HHS remained an infrequent diagnosis despite the use of BHB analysis is of interest. It would imply that lethal HHS is unusual.

Similarly to DKA, BHB analysis did not have an impact on the number of deaths by hypothermia as these remained stable during the study period. There was an increase in the proportion of BHB analyses in the hypothermia cases, but not to the same extent as in DKA and AKA. It is possible, though, that BHB analysis aided in the establishment of cause of death in individual cases of hypothermia. The diagnosis of hypothermia, similarly to that of DKA, was also better substantiated with BHB analysis.

When a new analysis method is introduced, one consequence may be analysis results that cannot be explained in detail. The lack of a good database for a gold standard provides a particular challenge in forensic medicine. New routines also require a change of attitude [25]. In our study, the diagnosis of acidosis NOS may reflect these problems. In our study, the number of unspecific acidosis cases (acidosis NOS) increased as the number of BHB analyses increased. The exact cause of the ketoacidosis in these cases cannot be concluded from this study. Probably at least some of them were DKA or AKA cases that for some reason were diagnosed only as acidosis. There also seemed to be a slight decrease in acidosis NOS cases in 2019 compared to 2018 in spite of the increasing analysis rate for BHB. Whether this trend will continue cannot be concluded here. However, it is possible that this decline of acidosis NOS diagnoses in 2019 was the effect of a greater understanding of the importance of BHB and an indication of an increased tendency to associate an augmented BHB concentration with an underlying condition, such as diabetes or alcoholism.

The use of the diagnosis of acidosis NOS also raises the question whether this is a sufficient diagnosis to pass as the only cause of death. Acidosis may develop for a number of reasons, both natural and unnatural causes [12,16]. If only acidosis NOS is marked on the death certificate, the case will automatically be labelled as death by natural causes, even though the condition may have been caused for example by a trauma. The forensic scientific community and those responsible for the cause-of-death statistics need to have an approach for how to handle this dilemma if the underlying reason for the acidosis cannot be concluded from the autopsy and ancillary investigations.

As can be seen from Figure 3, there was a large increase in the number of BHB analyses during the latter part of the study period, starting in 2016. In spite of this increase, the proportion of elevated (BHB 50–249 μg/g) and ketoacidotic results (BHB ≥ 250 μg/g) remained at more than 50% until 2019. Even in 2019, when the normal BHB results (BHB < 50 μg/g) constituted the majority of all analysis results, a larger number of BHB results between 50 and 249 μg/g were detected thanks to the increased analysis rate.

Concerning the cases with a BHB result ≥ 250 μg/g, there is a noticeable drop in the proportion of cases with a non-acidotic cause of death in 2019. The proportion of cases with a BHB result ≥ 250 μg/g that had a non-acidotic cause of death was much larger in 2016 through 2018. The change in 2019 may reflect an increased confidence in the use of BHB analysis and an increased proneness by the forensic pathologists to rely on it when establishing the cause of death.

Concerning the ten most frequent non-acidotic diagnoses among the cases with a BHB concentration ≥ 250 μg/g, it should be noted that the number of cases per diagnosis are few. The cases also reflect a total of the entire seven-year study period. Thus, possible changes during the study period are not detected and overall, the numbers must be interpreted with caution. It is nevertheless noteworthy that chronic alcoholism was the second most frequent diagnosis among the ten most frequent non-acidotic diagnoses. This might indicate that in these cases, the BHB result was overlooked and that these cases should actually have been diagnosed as AKA. No conclusions overall can be made concerning the correctness of the set cause of death in this study, as its purpose is to study the number and use of various diagnoses by the forensic pathologists after the introduction of BHB analysis. Yet, it can be noted that in addition to chronic alcoholism, three further diagnoses (coronary atherosclerosis, old myocardial infarction, and cardiac hypertrophy) were also of a chronic nature even though the existing ketoacidosis would have offered a more acute cause of death. There were also eight cases in which the cause of death was marked as unknown, in spite of a BHB concentration ≥ 250 μg/g. These results may be an indication that it takes time for a new method to become established, in this case BHB analysis, before it is fully accepted and recognised. It has also been suggested that forensic pathologists, in the absence of known diabetes or alcoholism, may underestimate the importance of a biochemically verified ketoacidosis [26].

## 5. Conclusions

It has been postulated that post-mortem biochemical markers are viewed with some scepticism in the field of forensic pathology [3,27,28,29]. Our study illustrates the concrete effects of BHB analysis on the establishment of cause of death and underscores the importance of this biochemical analysis. The introduction of BHB analysis led to a large increase in the number of diagnosed cases of AKA in the Swedish forensic autopsy material. Thus, BHB analysis was vital for this diagnosis. Surprisingly, the number of deaths from chronic alcoholism did not decrease simultaneously but remained stable. BHB analysis may therefore, besides being instrumental in the diagnosis of AKA, have had the additional effect of highlighting the lethal nature of chronic alcoholism.

In contrast to AKA and chronic alcoholism, BHB analysis did not affect the number of deaths from diabetes and related acute conditions, nor did it affect the number of deaths from hypothermia. However, BHB analysis most likely has improved the quality of these diagnoses as an additional indicator of these conditions.

The number of deaths from acidosis NOS increased as the number of BHB analyses increased. This effect of introducing BHB analysis raises the question of the correct diagnosing of cases with a high BHB concentration but without an obvious cause for the elevation. The number of acidosis NOS cases may be a consequence of the challenges of implementing biochemical analyses in the practical forensic work as previously mentioned [25].

In spite of a very large increase in the number of BHB analyses, the proportion of elevated and ketoacidotic results remained high during the entire study period. During the last year of the study period, the number of ketoacidotic cases that were not considered to have a ketoacidotic cause of death dropped. This may reflect the time period that is needed for a method to be fully accepted and applied in practical work.

Overall, BHB analysis proved important for the establishment of cause of death in the Swedish forensic autopsy practice. It enabled a new diagnosis to be made (AKA) and improved the quality of other diagnoses, such as DKA and hypothermia. BHB analysis is therefore an important and very useful tool in practical establishment of cause of death.

## Figures and Tables

**Table 1:** All cases analysed for BHB per year.

Year	DKA ^1^ (*n*)	AKA ^2^ (*n*)	HHS ^3^ (*n*)	Hypothermia (*n*)	Acidosis NOS ^4^ (*n*)	Diabetes NOS ^4^ (*n*)	Chronic Alcoholism (*n*)	Others (*n*)	Total (*n*)
2013	2	2	0	0	1	0	1	7	13
2014	5	10	0	0	0	0	1	12	28
2015	6	7	0	0	0	0	0	6	19
2016	21	22	0	6	3	1	15	55	123
2017	56	43	1	11	14	2	40	264	431
2018	63	51	4	23	25	4	55	644	869
2019	62	66	3	32	19	2	84	1149	1417
Total (*n*)	215	201	8	72	62	9	196	2137	2900

Notes: ^1^ diabetes ketoacidosis, ^2^ alcoholic ketoacidosis, ^3^ hyperosmolar hyperglycaemic state, ^4^ not otherwise specified.

**Table 2:** Other cases (not analysed for BHB) per year.

Year	DKA ^1^ (*n*)	AKA ^2^ (*n*)	HHS ^3^ (*n*)	Hypothermia (*n*)	Acidosis NOS ^4^ (*n*)	Diabetes NOS ^4^ (*n*)	Chronic Alcoholism (*n*)	Total (*n*)
2013	56	1	2	50	0	9	99	217
2014	44	0	3	36	0	5	91	179
2015	47	1	4	35	0	7	106	200
2016	38	2	3	39	0	10	91	183
2017	11	1	1	27	1	1	69	111
2018	4	3	0	35	3	5	55	105
2019	2	0	0	25	1	1	45	74
Total (*n*)	202	8	13	247	5	38	556	1069

Notes: ^1^ diabetes ketoacidosis, ^2^ alcoholic ketoacidosis, ^3^ hyperosmolar hyperglycaemic state, ^4^ not otherwise specified.

**Table 3:** Total (Table 1 and Table 2 together).

Year	DKA ^1^ (*n*)	AKA ^2^ (*n*)	HHS ^3^ (*n*)	Hypothermia (*n*)	Acidosis NOS ^4^ (*n*)	Diabetes NOS ^4^ (*n*)	Chronic Alcoholism (*n*)	Others (*n*)	Total (*n*)
2013	58	3	2	50	1	9	100	7	230
2014	49	10	3	36	0	5	92	12	207
2015	53	8	4	35	0	7	106	6	219
2016	59	24	3	45	3	11	106	55	306
2017	67	44	2	38	15	3	109	264	542
2018	67	54	4	58	28	9	110	644	974
2019	64	66	3	57	20	3	129	1149	1491
Total (*n*)	417	209	21	319	67	47	752	2137	3969

Notes: ^1^ diabetes ketoacidosis, ^2^ alcoholic ketoacidosis, ^3^ hyperosmolar hyperglycaemic state, ^4^ not otherwise specified.

## Data Availability

Restrictions apply to the availability of these data. Data were obtained from the National Board of Forensic Medicine and are available from the authors with the permission of the National Board of Forensic Medicine.
